# Pre-infection transcript levels of *FAM26F* in peripheral blood mononuclear cells inform about overall plasma viral load in acute and post-acute phase after simian immunodeficiency virus infection

**DOI:** 10.1099/jgv.0.000632

**Published:** 2016-12-15

**Authors:** Aneela Javed, Nicole Leuchte, Gabriela Salinas, Lennart Opitz, Christiane Stahl-Hennig, Sieghart Sopper, Ulrike Sauermann

**Affiliations:** ^1^​ Deutsches Primatenzentrum GmbH, Leibniz-Institut für Primatenforschung, Unit of Infection Models, Göttingen, Germany; ^2^​ Transcriptome and Genome Analysis Laboratory (TAL), Faculty of Medicine, University of Göttingen, Göttingen, Germany; ^3^​ Tumor Immunology Lab, Hematology and Oncology, Medical University Innsbruck and Tyrolean Cancer Research Institute, Innsbruck, Austria

**Keywords:** SIV, HIV, FAM26F, innate immune response, IFN-gamma, acute infection

## Abstract

CD8^+^ cells from simian immunodeficiency virus (SIV)-infected long-term non-progressors and some uninfected macaques can suppress viral replication *in vitro* without killing the infected cells. The aim of this study was to identify factors responsible for non-cytolytic viral suppression by transcriptional profiling and to investigate their potential impact on SIV replication. Results of microarray experiments and further validation with cells from infected and uninfected macaques revealed that *FAM26F* RNA levels distinguished CD8^+^ cells of controllers and non-controllers (*P*=0.001). However, *FAM26F* was also expressed in CD4^+^ T-cells and B-cells. *FAM26F* expression increased in lymphocytes after *in vitro* IFN-γ treatment on average 40-fold, and *ex vivo FAM26F* RNA levels in peripheral blood mononuclear cells correlated with plasma IFN-γ but not with IFN-α. Baseline *FAM26F* expression appeared to be stable for months, albeit the individual expression levels varied up to tenfold. Investigating its role in SIV-infection revealed that *FAM26F* was upregulated after infection (*P*<0.0008), but did not directly correlate with viral load in contrast to *MX1* and *CXCL10*. However, pre-infection levels of *FAM26F* correlated inversely with overall plasma viral load (AUC) during the acute and post-acute phases of infection (e.g. AUC weeks post infection 0–8; no AIDS vaccine: *P*<0.0001, Spearman rank correlation coefficient (rs)=−0.89, *n*=16; immunized with an AIDS vaccine: *P*=0.033, rs=−0.43; *n*=25). *FAM26F* transcript levels prior to infection can provide information about the pace and strength of the antiviral immune response during the early stage of infection. *FAM26F* expression represented, in our experiments, one of the earliest prognostic markers, and could supplement major histocompatibility complex (MHC)-typing to predict disease progression before SIV-infection.

## Introduction

Infection with the human immunodeficiency virus (HIV-1) is one of the world's most significant public health challenges. In contrast to most untreated HIV-infected patients, a small subset of patients, called long-term non-progressors (LTNPs) can maintain low or undetectable viraemia and physiological CD4^+^ T-cell levels and remain clinically asymptomatic for years (Deeks & Walker, 2007; Poropatich & Sullivan, 2011; Sheppard *et al.*, 1993). CD8^+^ lymphocytes are well documented to have a pivotal role in direct control of viral replication, e.g. by classical major histocompatibility complex (MHC) class I restricted antigen-specific cytotoxic T lymphocyte (CTL) effector mechanism and non-classical CD8 T-cell mediated non-cytolytic antiviral response (CNAR) (Mackewicz *et al.*, 2003; Walker *et al.*, 1986, 1991). CNAR refers to the property of CD8^+^ T-lymphocytes to suppress HIV-1 replication in acutely infected CD4^+^ cells without killing them (Levy, 2003; Stranford *et al.*, 1999; Vella & Daniels, 2003; Walker *et al.*, 1991; Wiviott *et al.*, 1990). High CNAR has been reported to correlate with an asymptomatic healthy clinical state and long-term survival of HIV-infected individuals (Blackbourn *et al.*, 1996; Castelli *et al.*, 2002; Greco *et al.*, 1998; Mackewicz *et al.*, 1991; Yuan *et al.*, 2009).

Whole transcriptome analyses have been performed to identify the gene(s) mediating CNAR. The first study involved a pair of HIV-1 discordant identical twins (Diaz *et al.*, 2003). Another study included four CNAR-positive and four CNAR-negative HIV-infected individuals (Martinez-Mariño *et al.*, 2007). There was no overlap between the genes identified in these two reports. Comparison of gene expression profiles from CD8^+^ cells of three children with differential CNAR identified more than 7000 differentially expressed genes belonging to diverse cellular processes (Katz *et al.*, 2011). While there was a significant overlap in differential gene expression between this and the two former studies, unfortunately no gene represented a strong candidate gene for CNAR.

Simian immunodeficiency virus (SIV)-infected macaques represent the most accepted model for HIV-infection. Our previous studies showed that CNAR can be detected over years in SIV-infected LTNPs and is inversely correlated with plasma viral load (Javed *et al.*, 2015). The present study focused on the identification of factors associated with non-cytolytic viral suppression using CD8^+^ cells displaying high CNAR activity and CD8^+^ cells that lack CNAR. Microarray experiments were performed, notably including CNAR(+) cells from infected and uninfected macaques. The most significantly differentially expressed gene, *FAM26F*, appeared to be related to immune activation, which provided the rationale for further studies. Investigations into a potential role in SIV-infection revealed that the transcript levels of *FAM26F* were predictive of overall plasma viral load during acute and post-acute phase of SIV-infection.

## Results

### Gene expression profile comparison of CNAR(+) and CNAR(−) CD8 cells

Global gene expression profiles were determined from CD8^+^ cells of CNAR(+) and CNAR(−) SIV-infected LTNPs and uninfected macaques after performing a viral inhibition test. Uninfected macaques were included to minimize the effects that might potentially be related to long-term SIV-infection and distinguishes this work from previously published experiments in HIV-infected humans (Diaz *et al.*, 2003; Katz *et al.*, 2011; Killian *et al.*, 2013; Martinez-Mariño *et al.*, 2007). Comparison between CNAR(−) versus CNAR(+) animals revealed 143 differentially regulated genes (differential expression over twofold, *P*<0.1), which belonged to diverse biological processes (Table S1, Fig. S1, available in the online Supplementary Material). Only 14 genes were upregulated in CNAR(+) animals.

Furthermore, except for glutamate-ammonia ligase (*GLUL*) none of the genes was previously identified as differentially regulated in CNAR(+) individuals (Diaz *et al.*, 2003; Katz *et al.*, 2011; Martinez-Mariño *et al.*, 2007). Notably, no soluble factors such as chemokines or cytokines known to be associated with HIV or SIV replication were differentially expressed when combined data from SIV-infected and non-infected monkeys were analysed (Table S1).

Some of the downregulated genes in CNAR(+) CD8^+^ cells have already been investigated in HIV-infection. Lower expression of protein disulfide isomerase family A member 4 (*PDIA4),* dipeptidylpeptidase 4 (*DPP4/CD26*) or tumour necrosis factor (ligand) superfamily member 13b (*TNFSF13B/BAFF*) may contribute to inhibition of HIV in CD4^+^ T-cells but does not explain how this should affect anti-HIV/SIV CD8 T-cell function (Bernier *et al.*, 2013; Fan *et al.*, 2012; Gallina *et al.*, 2002; Jager *et al.*, 2012; Reiser *et al.*, 2012; Rotger *et al.*, 2011; Songok *et al.*, 2010; Stantchev *et al.*, 2012). Notably, many (26 % of downregulated, 46 % of upregulated) differentially expressed genes encode membrane proteins belonging to activation/signalling pathways and/or are involved in cell adhesion.

### Lower *in vitro* expression of *FAM26F* in CD8^+^ cells from CNAR(+) macaques

**Fig. 1.**
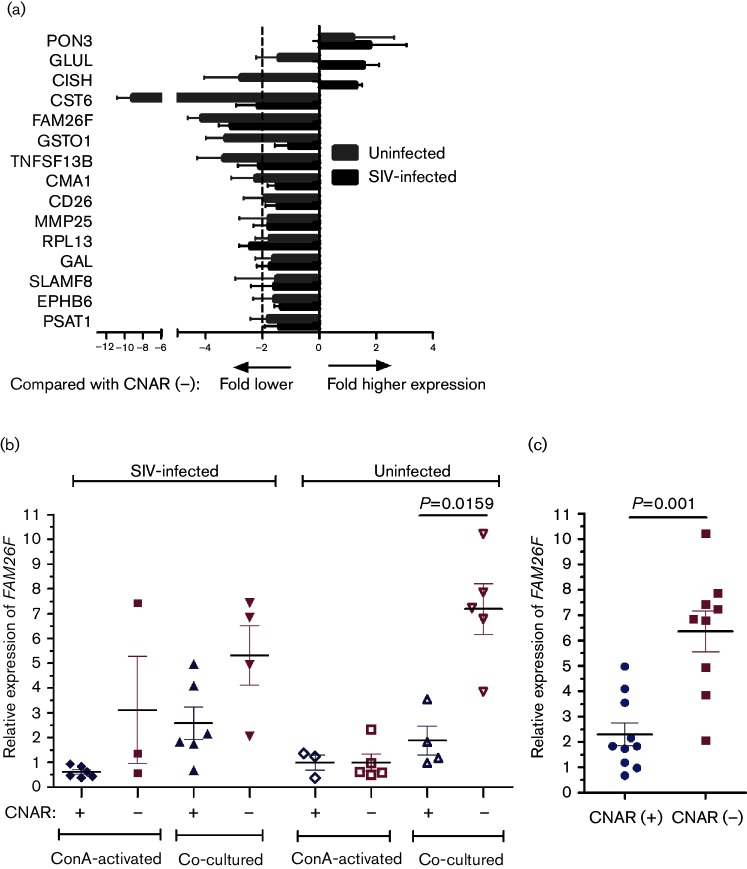
Differential expression of 15 genes in CD8^+^ cells from CNAR(+) animals compared with CNAR(−) animals. (a) RNA was extracted from re-isolated CD8^+^ cells of six CNAR(+) and four CNAR(−) SIV-infected LTNPS, as well as from four CNAR(+) and five CNAR(−) uninfected monkeys after the seventh day of co-culture with SIV-infected MHC-mismatched CD4^+^ cells. Fold difference in gene expression was calculated using the 2^−ΔΔ*C*_t_^ formula. Mean with standard deviation is shown. (b) Relative *FAM26F* RNA levels compared with the housekeeping gene *GAPDH* in CD8^+^ cells after overnight concanavalin A (ConA) activation and after day 7 of co-culture. (c) Relative *FAM26F* RNA levels in CD8^+^ cells from combined infected and uninfected CNAR(+) (*n*=10) and CNAR(−) (*n*=9) macaques in day 7 co-cultures. Mean, standard error of the mean and significant *P* values are depicted (Mann Whitney *U* test).

Differential expression of 15 genes from microarray data was further investigated by quantitative reverse transcription PCR (RT-PCR) in CD8^+^ cells from six CNAR(+) and four CNAR(−) LTNPS, as well as from four CNAR(+) and five CNAR(−) uninfected monkeys (Fig. 1a). Since CNAR has been ascribed to proteinase activity (Mackewicz *et al.*, 2003) we also included *CMA1*, which was found to be upregulated fourfold in microarray analysis of uninfected CNAR(+) animals (data not shown). For validation purposes, the same protocol as for generation of the samples for the microarray analysis was employed.

Four genes were found to be more than twofold downregulated in SIV-infected CNAR(+) animals, and six genes in CD8^+^ cells from uninfected CNAR(+) macaques as compared with the respective CNAR(−) animals (Fig. 1a). Finally, only *FAM26F* RNA levels were significantly lower in CD8^+^ CNAR(+) compared with CNAR(−) cells from uninfected macaques (*P*=0.0159, Mann Whitney test; Fig. 1b). Combined data from uninfected and infected macaques confirmed that *FAM26F* RNA levels were significantly lower in CD8^+^ cells from CNAR(+) macaques compared with those from CNAR (−) macaques (*P*=0.001, Mann Whitney test, Fig. 1c).

### Differential expression of *FAM26F* emerged after co-cultivation with SIV-infected CD4^+^ cells

Next, we investigated whether the differential transcript levels of *FAM26F* in CD8^+^ cells emerged as a consequence of co-cultivation with SIV-infected CD4^+^ cells during the viral inhibition test or whether these differences were present before the start of the experiment. *FAM26F* RNA levels did not differ significantly either in CD8^+^ cells after concanavalin A (ConA) stimulation before initiation of the co-cultures (*P*>0.4, Mann Whitney test; Fig. 1b) or in *ex vivo*-isolated CD8^+^ cells between CNAR(+) and CNAR(−) monkeys (*P*>0.2, Mann Whitney test; data not shown). However, upon co-cultivation of CD8^+^ T-cells with SIV-infected CD4^+^ T-cells, *FAM26F* RNA levels increased up to tenfold (Fig. 1b). The increase after co-cultivation was on average three to four times stronger in cells from CNAR(−) monkeys as compared with cells from CNAR(+) monkeys and resulted in a lower expression of *FAM26F* in CNAR(+) animals. These findings indicated that *FAM26F* might not necessarily be related to CNAR but to immune activation and provided the rationale for further investigations into *FAM26F* expression and its potential role as a prognostic marker in SIV-infection.

### Expression of *FAM26F* in lymphocytes

**Fig. 2.**
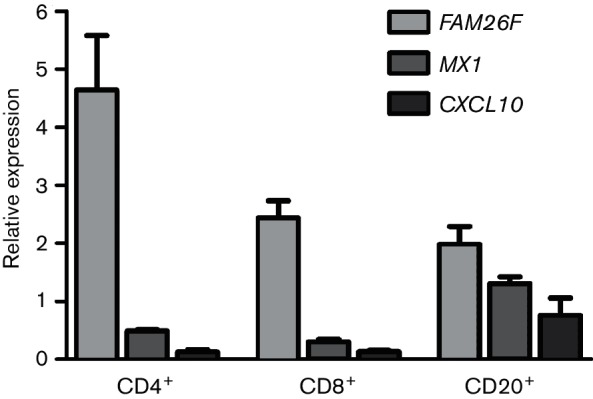
*FAM26F*, *MX1* and *CXCL10* expression in CD8^+^, CD4^+^ and CD20^+^ cells of four naive animals. Cell populations were isolated by magnetic bead purification, RNA was isolated and relative expression was quantified by RT-PCR. Mean and standard error of the mean are shown.

Expression of *FAM26F* was analysed by RT-PCR in three major lymphocyte populations, namely CD8^+^, CD4^+^ and CD20^+^ (B-cells), of four naive animals. RNA levels of the innate immune genes *MX1* and *CXCL10* were also measured. Maximum expression of *FAM26F* was observed in CD4^+^ cells. *MX1* and *CXCL10* were barely expressed, with highest levels in CD20^+^ cells (Fig. 2). The results demonstrate that *FAM26F* represents a gene that is well expressed in many lymphocyte populations. Because of this broad expression profile and the function of FAM26F via homophilic interactions (Ebihara *et al.*, 2010), we used peripheral blood mononuclear cells (PBMCs) in the further studies.

### *FAM26F* expression is linked to IFN-*γ* expression

To gain insight into *FAM26F* regulation, its expression was quantified after stimulation of PBMCs from eight LTNPs, and two and three uninfected monkeys with IFN-γ and IFN-α2, respectively.

**Fig. 3.**
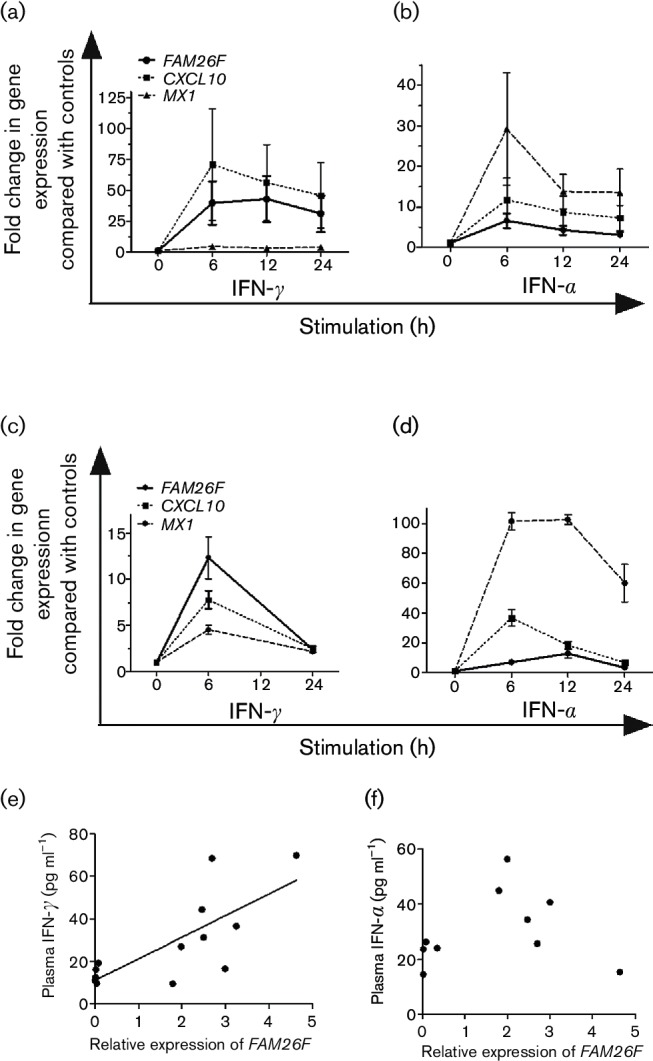
*FAM26F* expression is linked to IFN-γ pathway. IFN-γ was added to PBMCs from (a) eight LTNPs without and (c) two naive macaques with prior low-dose ConA activation (10 ng ml^−1^ overnight). IFN-α2 was added to PBMCs from (b) eight LTNPs without and (d) three naive macaques with prior low-dose ConA activation. At the time points indicated, RNA was extracted and fold differences in gene expression of *FAM26F*, *CXCL10* and *MX1* compared with controls not treated with interferons were determined. Mean and standard error of the mean are shown. (e) Plasma IFN-γ and (f) plasma IFN-α were quantified by ELISA while relative expression of *FAM26F* and *CXCL10* was quantified by RT-PCR in PBMCs. *FAM26F* expression correlated with plasma IFN-γ (*P*=0.0031, Pearson *r*=0.7508; *n*=13) but not with plasma IFN-α (*n*=10).

*MX1* and *CXCL10/IP-10* as interferon type I and type II regulated genes were also investigated. At 6 to 12 h after IFN-γ stimulation, *FAM26F* RNA increased on average 40-fold following kinetics similar to those of *CXCL10* (Fig. 3a). *MX1* RNA levels increased only fourfold upon IFN-γ stimulation (Fig. 3a). Six hours after IFN-α2 stimulation of PBMCs from LTNPs, *MX1* RNA levels increased on average 30-fold, whereas *FAM26F* and *CXCL10* RNA levels increased only 7- and 12-fold, respectively (Fig. 3b). Maximum increase of *FAM26F* transcripts occurred 6 h after IFN-γ stimulation in cells from uninfected monkeys pre-treated with low concentrations of ConA (10 ng ml^−1^) in contrast to IFN-α2 stimulation (Fig. 3c, d).

In addition, relative RNA levels of *FAM26F* were quantified in blood samples of macaques immunized with viral vectors. As with other genes of innate immunity, expression of *FAM26F* increased significantly within 24 h compared with pre-immunization levels (data not shown). In parallel, plasma IFN-γ and IFN-α were quantified by ELISA. *FAM26F* RNA correlated significantly with plasma IFN-γ (*P*=0.0031, Pearson *r*=0.7508; *n*=13) (Fig. 3e) but not with IFN-α (*P*=0.66, Pearson *r*=0.16, *n*=10) (Fig. 3f). In summary, our results suggest that *FAM26F* RNA levels are linked to the IFN-γ pathway.

### *FAM26F* expression increases after SIV-infection

**Fig. 4.**
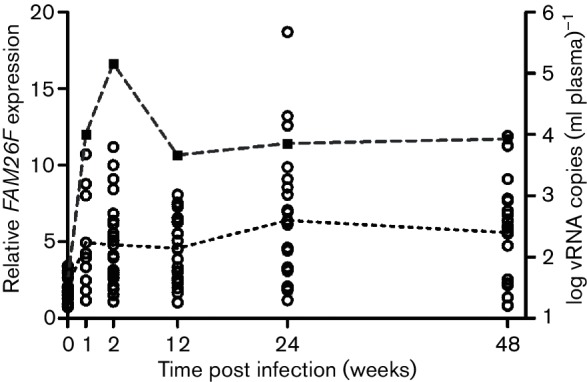
*FAM26F* gene expression after SIV-infection in relation to viral load. (a) Relative *FAM26F* transcript levels were determined at specified time points, before and after SIV-infection of rhesus macaques. Each time point shows data from 9 to 24 macaques. Expression of *FAM26F* (open circles) was significantly upregulated after SIV-infection as compared with its pre-infection values until the end of the study period (*P*<0.0008, Mann Whitney test). Log_10_ of the geometric mean of plasma viral RNA (vRNA) copies is shown as a dashed line and filled squares. Mean of relative *FAM26F* gene expression is depicted as a dotted line.

The expression pattern of *FAM26F* before and after infection was investigated in two independent AIDS vaccine experiments termed E1 and E2 and one infection study (E3) comprising a total of 51 animals (see Methods and Table S2). *FAM26F* RNA in PBMCs was quantified at defined time points along with *MX1* and *CXCL10*. In all experiments, *FAM26F* transcripts were significantly elevated after SIV-infection as compared with pre-infection values until the end of the study period (*P*<0.0008 from week 2 up to weeks 32–48 post infection, Mann Whitney test, data for E1; Fig. 4).

We also investigated whether *FAM26F* RNA levels could be related to plasma viral load. However, *FAM26F* RNA did not correlate with viral load (e.g. weeks 2, 12, 24, 48 post infection; Fig. S2a). In contrast, but in line with previous publications, *MX1* did so (*P≤*0.0016, Spearman rank correlation coefficient (rs)>0.66 at week 24 post infection as example; Fig. S2b) (Mussil *et al.*, 2011). Relative expression of *CXCL10* correlated with viral load in two of the three experiments at week 24 post infection (Fig. S2c).

### Pre-infection RNA levels of *FAM26F* correlate robustly with overall plasma viral load in acute and post-acute infection in unimmunized macaques

To study if the differential expression of *FAM26F* can be an early predictor of viral load of the chronic phase of infection, potential correlations between *FAM26F* RNA levels and viral load were investigated. Investigating the predictive potential of RNA levels at 2 weeks post infection (wpi) revealed that *FAM26F*, *MX1* and *CXCL10* were predictive of chronic phase set-point viral load only when peak viraemia correlated with the further course of infection (Fig. S3).

**Fig. 5.**
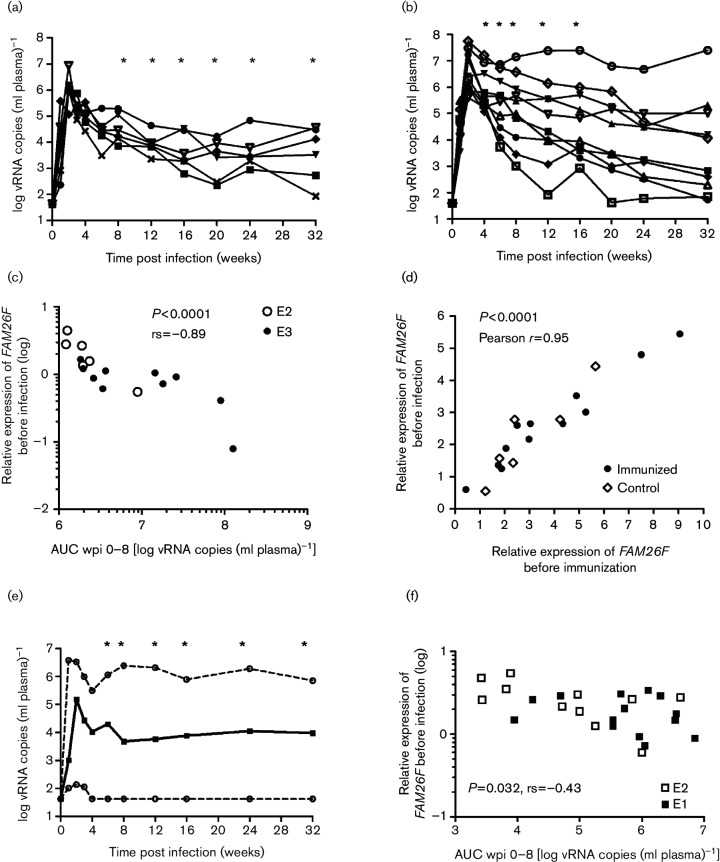
*FAM26F* gene expression at day of first SIV exposure correlates with the extent of early viral replication. (a) Log plasma viral RNA copies of unvaccinated macaques (*n*=6) from E2 are shown. Asterisks (*) mark significant correlation of *FAM26F* RNA levels at day of first SIV exposure with overall plasma viral load from infection to this time point. Significant correlations with overall viral load: AUC 0–8 to AUC 0–20: *P*=0.033, rs=−0.89; AUC 0–24 and AUC 0–32: *P*=0.017, rs=−0.94. (b) Log plasma viral RNA copies of unimmunized macaques (*n*=10) from E3 are shown. Asterisks (*) mark significant correlation of *FAM26F* RNA levels at day of first SIV exposure with overall plasma viral load from infection to this time point. Significant correlations with overall viral load: AUC 0–4: *P*=0.02, rs=−0.71; AUC 0–6: *P*=0.016, rs=−0.73; AUC 0–8: *P*=0.013, rs=−0.74; AUC 0–12 and AUC 0–16: *P*=0.029, rs=−0.68. (c) Relative expression of *FAM26F* in PBMCs at day of first SIV exposure correlated with overall plasma viral load 0–8 wpi in non-immunized macaques from two independent experiments (AUC wpi 0–8: E2: rs=−0.88, *P*=0.033, *n*=6; E3: rs=−0.74, *P*=0.013, *n*=10); *P* value and Spearman's correlation coefficient rs for the combined data set are shown in the figure. (d) Correlation of individual relative *FAM26F* RNA levels in PBMCs before immunization with those of day of first SIV exposure 10 months later in immunized (*n*=12) and unvaccinated control macaques (*n*=6). Significance and Pearson *r* are indicated in the figure. (e) Log plasma viral RNA copies of unvaccinated macaques (from E1 and E3, *n*=25) are shown as median (solid line) and upper and lower range (dashed lines). Asterisks (*) mark significant correlation of *FAM26F* RNA levels at day of first SIV exposure with overall plasma viral load from infection to this time point. Significant correlations with overall viral load: AUC 0–6: *P*=0.036, rs=−0.42; AUC 0–8: *P*=0.032, rs=−0.43; AUC 0–12: *P*=0.017, rs=−0.46; AUC 0–16: 0.017, rs=−0.48; AUC 0–24: *P*=0.014, rs=−0.45; AUC 0–32: *P*=0.029, rs=−0.43). (f) Relative expression of *FAM26F* in PBMCs at day of first SIV exposure correlated with overall plasma viral load 0–8 wpi (AUC wpi 0–8) in macaques from two experiments (E1, E2) immunized with an experimental AIDS vaccine (*n*=25). *P* value and Spearman rank correlation coefficient (rs) are shown.

Next, we investigated whether pre-infection transcript levels could have a prognostic value. Since immunization can influence early viral replication kinetics, we first analysed unvaccinated macaques. Pre-infection samples were available from E2 (Fig. 5a; *n*=6) and E3 (Fig. 5b; *n*=10). In each experiment, pre-infection RNA levels of *FAM26F* correlated inversely with overall (AUC) plasma viral load during the acute and post-acute phases of infection [e.g. area under curve (AUC) plasma viral load wpi 0–8; E2: rs=−0.88, *P*=0.019; E3: rs=−0.74, *P*=0.013; Fig. 5a, b]. Combining both data sets confirmed the highly significant inverse correlation of *FAM26F* expression with the overall plasma viral load during the early phase of infection (Fig. 5c; e.g. AUC viral load wpi 0–8: rs=−0.89, *P*<0.0001). Note that the overall viral load is mainly (albeit not exclusively) influenced by the peak and post-peak (e.g. 4 wpi) viral load so that RNA copy numbers at later time points add relatively little to the overall plasma viral load. Consequently, the pre-infection RNA levels can also correlate with AUC values earlier or later than 8 wpi depending on the experiment (Fig. 5a, b). For E2, pre-infection RNA levels of *FAM26F* correlated with overall plasma viral load from AUC 0–8 to AUC 0–32 wpi, which was the latest available time point for all six animals (Fig. 5a). In E3, AUC values from 0–4 to 0–16 wpi correlated with pre-infection *FAM26F* RNA levels (Fig. 5b).

Next we investigated whether the presence of MHC class I alleles known to be associated with slower disease progression had influenced the results. In each experiment (E2, E3) 50 % of the macaques carried Mamu-A1*001, which is in our experiments significantly associated with slow disease progression (Muhl *et al.*, 2002; Sauermann *et al.*, 2008). Combining the results from E2 and E3 showed that *FAM26F* RNA levels were associated with overall viral load (AUC 0–8) in both Mamu-A1*001-positive and -negative monkeys (Mamu-A1*001-positive: *P*=0.021, rs=0.81, *n*=8; Mamu-A1*001-negative: *P*=0.0004, rs=−0.9762, *n*=8; Fig. S4a). Furthermore, neither the relative gene expression of *FAM26F* (*P*=0.38; Fig. S4b) nor the total viral load (AUC 0–8, *P*=0.44, Mann Whitney test; Fig. S4c) differed significantly between the groups, excluding a systematic influence of the MHC genotype on our data set.

*CXCL10* RNA levels before infection correlated with overall plasma viral load only after combining both data sets and with a lower significance level (e.g. AUC wpi 0–8: rs=−0.72, *P*=0.0017; Fig. S4d). Pre-infection *MX1* RNA levels did not correlate with overall plasma viral load (Fig. S4e).

Pre-infection RNA levels of *FAM26F* did not correlate with plasma viral load later on. However, overall early plasma viral load (AUC wpi 0–8) correlated with chronic phase plasma viral RNA copies at wpi 12, 16, 24 and 32 (*r*>0.52, *P*<0.043, *n*=16; Pearson correlation for a linear relationship; Fig. S4f), which represented the latest available data for all non-immunized animals. Taken together, pre-infection RNA levels of *FAM26F* were robustly associated with overall plasma viral load in the early phase of infection, which was in turn associated with chronic phase viral load.

### Pre-infection *FAM26F* RNA levels correlate with extent of early viral replication in vaccinated macaques

A potential association of pre-infection gene expression of *FAM26F*, *CXCL10* and *MX1* with viral replication in macaques immunized with experimental AIDS vaccines from E1 and E2 was also investigated.

Immunization had a strong impact on early viral replication (E2: reduction of overall plasma vial load wpi 0–8 in vaccinees compared with controls >1 log, *P*=0.0057; E1: varying effects depending on the vaccine formulation; Tenbusch *et al.*, 2012). Strong associations were therefore not expected. In addition, *FAM26F* RNA levels could be analysed for E2 before the start of the immunization and 10 months later, on the day of first SIV exposure. Relative expression of *FAM26F* prior to immunization and infection correlated significantly (Pearson *r*=0.95, *P*<0.0001; Fig. 5d) indicating that expression was stable for months and that *FAM26F* expression levels on the day of first exposure were comparable to baseline.

In neither immunized macaques from E1 (available pre-infection samples: *n*=14) nor those from E2 (*n*=11) did relative expression of *FAM26F*, *MX1* and *CXCL10* before infection correlate with the extent of early viral replication. However, combining both data sets revealed that only RNA levels of *FAM26F* correlated with the extent of early viral replication (e.g. AUC 0–8, *FAM26F*: *P*=0.033, rs=−0.43; *n*=25; Fig. 5c, d). The association between pre-infection *FAM26F* RNA levels and overall plasma viral load was observed in the time intervals ranging from 0–4 to 0–32 wpi (Fig. 5e). Furthermore, combined data sets also revealed a correlation between pre-infection levels of *FAM26F* RNA and plasma viral RNA copies at weeks 12 and 24–26 post infection (Fig S5a). These results confirm an association of *FAM26F* RNA levels with chronic phase viral load (*P*<0.046, rs>−0.4) in immunized macaques. Neither pre-infection *CXCL10* (Fig. S5b) nor *MX1* (Fig. S5c) transcript levels correlated with overall early plasma viral load.

No significant association of early viral replication (AUC wpi 0–8) with chronic phase viral load was evident for E1 (Fig. S5d). However, in E2 it correlated with plasma viral load at weeks 24 and 32 (Pearson *r*>0.97, *P*<0.0001, *n*=11; Fig. S5e).

## Discussion

This study initially aimed at the identification of factors responsible for non-cytolytic viral suppression in SIV-infected rhesus macaques by transcriptional profiling. Since *in vitro* viral inhibition tests for identification of CNAR(+) and CNAR(−) animals showed that CD8^+^ T-cells of non-infected animals can display CNAR, CD8^+^ cells from both SIV-infected and uninfected macaques were investigated (Javed *et al.*, 2015). This two-pronged strategy increased the probability of identifying the differentially expressed genes that were not influenced by previous SIV-infection. After replicating microarray results of selected genes in 19 macaques, *FAM26F* RNA levels could be distinguished in CD8^+^ cells from CNAR(+) macaques compared with those from CNAR(−) animals. *FAM26F* RNA levels increased in CD8^+^ cells after co-cultivation with SIV-infected CD4^+^ cells, but no differences were evident in *ex vivo* isolated CD8^+^ cells or in CD8^+^ cells after ConA activation. Investigating *FAM26F* transcript levels in SIV-infection showed that they increased also after SIV-infection. Higher *FAM26F* RNA levels in CNAR(−) compared with CNAR(+) cells after a 7-day culture with SIV-infected cells could therefore be explained by the higher SIV replication and probably stronger immune activation in CNAR(−) compared with CNAR(+) cell cultures. If the interpretation that *FAM26F* might not be necessarily related to CNAR but to immune activation is correct, our results are in line with other attempts that failed to identify by microarray analysis a strong candidate gene contributing to CNAR (Diaz *et al.*, 2003; Katz *et al.*, 2011; Martinez-Mariño *et al.*, 2007). Perhaps conspicuous gene expression differences are not the hallmark that distinguishes CNAR(+) and CNAR(–) CD8^+^ T-cells but rather the equipment with distinct polymorphic genes. In this respect, a recent report has uncovered mechanisms underlying cell-contact-mediated CNAR (Lu *et al.*, 2016) by showing that particular regulatory CD8^+^ T-cells in carriers of KIR3DL1 and HLA-B : Bw4-80Ile can suppress viral replication in CD4^+^ T-cells. However, in the interaction of the specialized subset of CD8^+^ cells with CD4^+^ cells, *FAM26F* could play a role, and a low expression may contribute to a reduced activation of CD4^+^ T-cells.

Our aim was then to investigate whether this is of potential prognostic value. We found that *FAM26F* gene expression in PBMCs before infection correlated inversely with overall plasma viral load during acute and post-acute phases of infection. This was most robust in unvaccinated monkeys but an effect was still evident in vaccinated macaques and may indicate that a certain pre-activation status could contribute to limit viral replication during early infection. *FAM26F* is a tetraspanin-like membrane glycoprotein (Ebihara *et al.*, 2010) and shows sequence similarity to an ion transporter (NCBI gene information; www.ncbi.nlm.nih.gov/gene).

*FAM26F* can make homophilic interactions by which synapses between cells are potentially established (Ebihara *et al.*, 2010). To date, no other interacting partner is known (NCBI gene information; www.ncbi.nlm.nih.gov/gene). *FAM26F* expression was required on both natural killer (NK) cells and myeloid dendritic cells to activate NK cells, and the cytoplasmatic tail was required for directed signal transmission from bone marrow derived dendritic cells to NK cells (Ebihara *et al.*, 2010).

Detailed knowledge about its function is largely lacking, but differential *FAM26F* expression has been detected by several whole-transcriptome analyses. *FAM26F* expression was one of the top classifiers for prognosis or diagnosis in a wide range of clinical settings, most of them related to inflammation (Defamie *et al.*, 2008; Kim *et al.*, 2009; Pankla *et al.*, 2009; Shahzad *et al.*, 2010). Increased *FAM26F* expression was also associated with clearance of hepatitis C virus (Grimes *et al.*, 2013) and clinical benefit through immune-based MAGE-A3 therapy in melanoma patients (Ulloa-Montoya *et al.*, 2013).

Insight into the transcriptional regulation of *FAM26F* is beginning to emerge. Its expression is upregulated by numerous signalling pathways that are likely to act synergistically (Chmielewski *et al.*, 2014). *FAM26F* expression on dendritic cells increases after TLR3 via polyI : C or TLR4 stimulation (Chmielewski *et al.*, 2014; Ebihara *et al.*, 2010; Lee *et al.*, 2014), upon IFN-β exposure (Lee *et al.*, 2014), by stimulating the dectin-1 pathway (Chiba *et al.*, 2014) and after murine cytomegalovirus (MCMV) infection (Manh *et al.*, 2013). Furthermore, deletion of IFNRA1 (IFN-α and IFN-β receptor) in mice resulted in abrogation of *FAM26F* induction by polyI : C (Kasamatsu *et al.*, 2014). Depending on the initial signal, IRF-3 and TICAM-1/TRIF (Ebihara *et al.*, 2010) or IRF-5 (Chiba *et al.*, 2014) are required, since their deletion results in loss of or markedly reduced *FAM26F* expression in dendritic cells/macrophages and impaired activation and cytolytic function of NK cells. These pathways can merge in STAT1 activation, which probably switches on *FAM26F* transcription (Chmielewski *et al.*, 2014; Lee *et al.*, 2014).

*FAM26F* expression is also linked to the IFN-γ pathway. We found that *FAM26F* gene expression in macaques correlated with plasma IFN-γ and increased in lymphocyte cultures after addition of IFN-γ. IFN-α could also stimulate *FAM26F* transcription. However, its effect was moderate and is likely to depend on the pre-activation status of the lymphocytes. In other species, IFN-γ alone (Brown *et al.*, 2010; Chmielewski *et al.*, 2014) or IFN-γ in combination with lipopolysaccharide or IFN-β (Chmielewski *et al.*, 2014; Zhang *et al.*, 2010) also leads to an increase of *FAM26F* RNA in different immune cells. Depletion of IFN-γ with a monoclonal antibody in human patients resulted in downregulation of *FAM26F* in blood cells (Welcher *et al.*, 2015). In addition, direct knock-out of *FAM26F* in mice resulted in significantly reduced initial production of IFN-γ after polyI : C treatment and reduced elimination of tumour cells (Kasamatsu *et al.*, 2014). These and our results indicate that *FAM26F* transcription is stimulated by IFN-γ but that *FAM26F* itself can also augment IFN-γ responses.

**Fig. 6.**
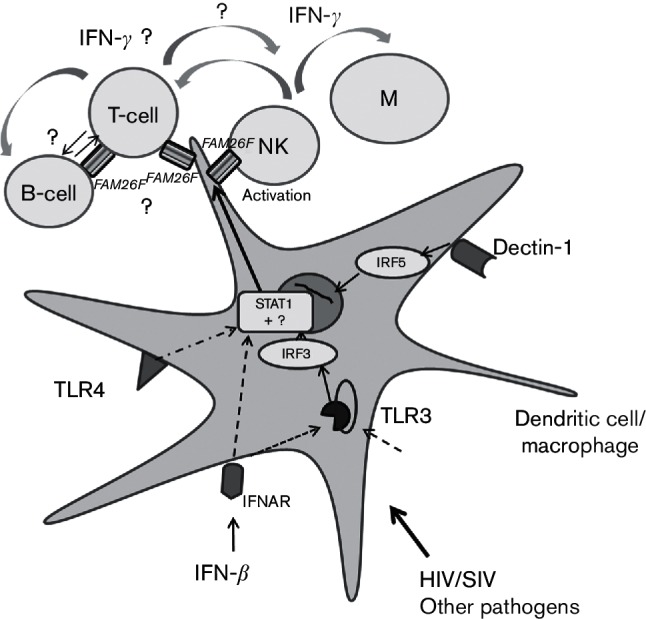
Schematic view illustrating current knowledge and hypothetical role of *FAM26F* in immune response induction. Activation of dendritic cells/macrophages via IFN-*α*/*β* receptor (IFNAR), TLR3, TLR4 and/or dectin-1 and pathogens results in increased *FAM26F* expression probably through activation of STAT1 (Chiba *et al.*, 2014; Chmielewski *et al.*, 2014; Ebihara *et al.*, 2010; Kasamatsu *et al.*, 2014; Lee *et al.*, 2014; Zhang *et al.*, 2010). Deletion of *IRF-3* or *IRF-5* reduces *FAM26F* expression in mice (Chiba *et al.*, 2014; Ebihara *et al.*, 2010). Expression of *FAM26F* contributes to early IFN-γ secretion and tumour reduction by NK cells (Kasamatsu *et al.*, 2014). The effect of *FAM26F* expression in T-cells, B-cells and other immune cells in terms of further augmentation of an IFN-γ response through *FAM26F-*mediated cell–cell contact is hypothetical and requires further investigation.

Furthermore*, FAM26F* is rapidly induced after stimulation as shown in the *in vitro* assays, *in vivo* studies in human PBMCs after vaccination (Matsumiya *et al.*, 2014) and in mice 5 h after staphylococcal enterotoxin B exposure (Ferreyra *et al.*, 2014) and represents therefore an early/immediate IFN-induced gene. Current knowledge on regulation of *FAM26F* transcription and action as well as its potential effects on T-cells is shown in Fig. 6.

So far, detailed investigations have focused on the interaction of FAM26F in dendritic cells with NK cells for cancer therapy (Chiba *et al.*, 2014; Ebihara *et al.*, 2010; Kasamatsu *et al.*, 2014). Investigations concerning its role in activation of T-cells and adaptive immune responses are still lacking. The present study showed that pre-infection RNA levels of *FAM26F* in blood lymphocytes represent a strong prognostic marker for viral replication during the acute and post-acute phase of SIV-infection. Notably, *FAM26F* gene expression did not correlate with peak viraemia but with the overall viral load during the early phase of infection. This phase includes reduction of viraemia by innate immune system and cytolytic responses mediated by adaptive immunity. Assuming that *FAM26F* plays an important role in the initiation of IFN-γ-governed adaptive and innate immune responses, high *FAM26F* expression before infection could contribute to or facilitate a fast activation of a collection of antiviral activities that limit early viral replication and determine further disease progression. Given that early innate responses are already inhibited during the first days of SIV-infection, a certain pre-activation status may be crucial to compensate for some of these effects (Barouch *et al.*, 2016). IFN-γ plays a key role in macrophage, NK cell and CD8^+^ T-cell activation, and is recognized as a hallmark of the subset 1 of helper T-cells (Th1). Moreover, SIV-specific CTLs are detectable at 2 weeks post infection and peak 1 week later (Allen *et al.*, 2000; O'Connor *et al.*, 2012). Plasma viral loads in SIV-infected macaques that control viral replication and those that do not start to diverge as early as 4 weeks post infection and differ significantly by 8 weeks post infection (Mudd & Watkins, 2011). During this early phase of infection the immune system can become irreversibly damaged. Among the pronounced effects is the massive depletion of gut-associated lymphoid tissue (GALT) CD4^+^ T-cells (Barouch *et al.*, 2016; Lim *et al.*, 1993; Mattapallil *et al.*, 2005; Schneider *et al.*, 1995). The acute phase thus represents a critical stage in HIV/SIV-infection, and the extent of early viral replication shapes the further course of infection. Albeit formally not proven, our results highlight that IFN-γ responses are of prominent importance for early control of viral replication.

Our results also have practical implications. *FAM26F* transcript levels represented in our experiments one of the earliest prognostic markers for disease progression after SIV-infection. Furthermore, the individual transcript levels differed significantly, and in the experimental setting, *FAM26F* expression at baseline appeared to be stable for months. In humans, polymorphisms close to *FAM26F* in the promotor region have been identified and are presumably linked to basal expression differences (Julià *et al.*, 2014; Lee *et al.*, 2014). The stable differences in *FAM26F* expression at baseline suggest that differential *FAM26F* expression may also be genetically determined in macaques. Since *FAM26F* expression is independent of the MHC genotype, it has a strong potential to supplement MHC-typing as a means to predict disease progression after SIV-infection and to evenly allocate macaques with similar immunobiological profile among the different experimental groups.

Assuming that *FAM26F* gene expression level is at least a hallmark for the differential speed and spread of early IFN-γ-guided immune responses, future investigations into its role on the initiation of innate and adaptive immune responses are pivotal. In addition, its potential role in local amplification of an immune response by cell–cell contact shall be a subject for further investigation and may also be important in the context of other viral infections. Importantly, appropriate tools to investigate FAM26F protein expression in humans and macaques need to be established.

## Methods

### Animal experiments.

Blood samples of 24 macaques of Indian origin were derived from an AIDS vaccine study described previously (experiment 1, E1) (Tenbusch *et al.*, 2012). An additional 18 macaques were from an AIDS vaccine study (experiment 2, E2) that included three groups, namely one control group and two vaccine groups consisting of six animals each (unpublished results). Following the immunization period, macaques were subjected weekly to low-dose intrarectal (i.r.) challenges with 120 TCID_50_ of SIVmac251 until >100 viral RNA copies (ml plasma)^−1^ were detected. Further details regarding the virus and the challenge procedure have been described previously (Tenbusch *et al.*, 2012). The third group consisted of 10 naive macaques (experiment 3, E3) that had been pre-exposed to escalating low doses of a SIVmac variant but had remained uninfected. After an 18-week waiting period, they became infected with SIVmac251 using the same virus stock and the same repeated low-dose i.r. challenge protocol as described above for E2. One to six inoculations were required to infect the monkeys not pretreated with an AIDS vaccine. The number of inoculations did not differ between E2 and E3 (log rank test, *P*=0.75). The LTNPs used for this study have been described before (Javed *et al.*, 2015) The LTNP status was defined as survival post SIV-infection for more than 1 year and plasma viral RNA copies below the detection limit of our assay or as a survival of the SIV-infection more than 3 years post infection (Javed *et al.*, 2015). All macaques were MHC-typed using standard techniques (Muhl *et al.*, 2002; Sauermann *et al.*, 2008).

### Ethical statement.

Rhesus macaques of Indian origin were housed at the German Primate Centre under standardized conditions according to the national and European Union guidelines on the use of non-human primates in biomedical research. The animal experiments were approved by the Lower Saxony State Office for Consumer Protection and Food Safety and performed with the project licences AZ 33.14-4502-04-017/07, AZ 33.14-42502-04-072/08, 33.14-42502-04-11/0626 and AZ 33.9-42502-04-12/0820.

The macaques had constant access to water and were fed twice a day with monkey dry food supplemented with fresh fruits and foraging or task-oriented feeding methods (e.g. treats, food puzzle). Physical examinations, bleedings and immunizations were carried out under ketamine anaesthesia [10 mg (kg body weight)^−1^]). In cases of suffering predefined by a scoring system of termination criteria that was approved by the external ethics committee and corresponds to the IACUC endpoint guidelines, monkeys were humanely euthanized in deep anaesthesia by an overdose of pentobarbitone sodium (Narcoren; Merial).

### Microarray experiment.

PBMCs were isolated through density gradient centrifugation (lymphocyte separation medium; PAA Laboratories) from 18 ml citrated blood of two CNAR(+) SIV-infected LTNPs, two CNAR(−) SIV-infected slow progressors (total survival of SIV-infection: 115 and 342 weeks post infection), and two CNAR(+) and two CNAR(−) non-infected monkeys, respectively. CD8^+^ lymphocytes were purified using the CD8^+^ isolation kit (Miltenyi). CD4^+^ cells from uninfected macaques were isolated from 9 ml blood using the CD4^+^ isolation kit (Miltenyi). CD8^+^ and CD4^+^ cells were re-suspended in complete RPMI medium [RPMI 1640 (PAN Biotech,), supplemented with 20 % FBS (PAN Biotech), 100 U penicillin ml^−1^ (PAN Biotech) and 100 µg streptomycin ml^−1^ (PAN Biotech)] and stimulated overnight with ConA (10 µg ml^−1^; SERVA Electrophoresis). CD4^+^ cells were infected with SIVmac239 as described previously (Ochieng *et al.*, 2009) and re-suspended in 1.5 ml complete RPMI medium. ConA-activated CD8^+^ cells were washed twice and re-suspended in 2 ml complete RPMI medium supplemented with 100 U recombinant human IL-2 ml^−1^ (PeproTech) and co-cultured with SIV-infected CD4^+^ cells. Fifty percent of the medium was replaced on days 3 and 6. On day 7, CD4^+^ cells were re-isolated with the CD4^+^ cell isolation kit (Miltenyi). Flow-through containing CD8^+^ cells was collected, centrifuged and re-suspended in 300 µl RNAprotect Cell Reagent (Qiagen) and frozen at −80 °C until further use.

RNA was isolated using the RNeasy Mini kit (Qiagen), digested with DNase (Qiagen) and purified again using the RNAeasy Mini kit. Quantity, purity and integrity were checked using a NanoDrop (Agilent Technologies) and an Agilent 2100 Bioanalyzer. cRNA synthesis, amplification and labelling were performed with 1 µg RNA using ‘Low RNA Input Linear Amplification kit PLUS, One Color’ (Agilent Technologies) and the Agilent ‘RNA One Color Spike-In kit’ following the manufacturer's instructions. Quality, quantity and efficiency of the labelling were checked with the Agilent 2100 Bioanalyzer and NanoDrop NS-1000. The fragmented cRNA of each sample was hybridized on 4×44 K monkey arrays (Agilent Technologies) in an ozone free environment (Hybridization Oven, Agilent). Each RNA sample was tested in duplicate. Washing and staining of the arrays were performed according to the manufacturer's recommendations. Cy3 intensities were detected by one-colour scanning using an Agilent microarray scanner at 5 µm resolution. Scanned image files revealed no artefacts upon visual inspection.

### Viral inhibition assay and re-isolation of CD8^+^ T-cells.

Samples from CNAR(+) and CNAR(−) macaques as well as the samples from which we re-isolated the CD8^+^ cells were from viral inhibition assays described in a previous study on CNAR (Javed *et al.*, 2015). Briefly, for the viral inhibition assays, CD4^+^ T-cells from SIV-uninfected, MHC-mismatched donors and CD8^+^ T-cells from SIV-infected and uninfected experimental animals were used. CD4^+^ T-cells and CD8^+^ T-cells were isolated from PBMCs by the magnetic bead separation technique for nonhuman primates (MACS) (Miltenyi Biotech) and activated with ConA in complete RPMI 1640 medium for 24 h. CD4^+^ cells were infected with SIVmac239 at an m.o.i. of 0.001 TCID_50_. *In vitro* infected CD4^+^ T-cells were either cultured alone (controls) or co-cultured with ConA-activated CD8^+^ cells from experimental animals in duplicate or triplicate at a 2 : 1 cell input ratio. Supernatants were collected on days 5 and 7 for viral RNA isolation. CD8^+^ cells were re-isolated at day 7 post infection by MACS techniques and frozen in RNAprotect Cell Reagent (Qiagen) until further use. Macaques were regarded as CNAR(+) when CD8^+^ cells inhibited viral replication >50 fold (Javed *et al.*, 2015).

### Cellular RNA extraction and cDNA synthesis.

For RT-PCR quantification of selected genes, cellular RNA from CD8^+^ T-cells was isolated by using the RNeasy Mini kit (Qiagen). Quality of RNA was checked by using an Agilent 2100 Bioanalyzer and average RIN (RNA integrity number) values for most samples were greater than 8.5. One hundred nanograms RNA was reverse transcribed using the Quantitect Reverse Transcription kit (Qiagen) and random primers. A no-reverse-transcriptase (NRT) reaction was included to ensure that there was no genomic DNA contamination. Total cellular RNA was isolated from whole blood stored in PAXgene Blood RNA tubes (BD Diagnostics) using the PAXgene Blood RNA kit (Qiagen). RNA was reverse transcribed by using the QuantiTect Reverse Transcription kit (Qiagen).

### Real-time PCR quantification of cellular genes.

Expression of cellular genes was quantified by RT-PCR in an ABI Prism 7500 cycler. Reactions were performed in 96 well MicroAmp Optical Reaction Plates (Applied Biosystems). Each 25 µl reaction mixture contained 12.5 µl Immomix (Bioline), 6.6 µl 10× ROX (Bioline), 0.5 µl SYBR Green (Lonza), 2 µM each of forward and reverse primers, and 1 µl of 1 : 5 diluted cDNA products. Cycling conditions were as follows: initial denaturation at 95 °C (7 min) followed by 40 cycles at 95 °C (15 s) and 57 °C (33 s). An additional melting/dissociation step was included to monitor amplification specificity. NRT controls were performed. Non-template controls and a cDNA as positive control were included in each PCR run. Primer sequences (Table 1) for validation of results of microarray experiments were designed using NCBI primer3online software (http://www.ncbi.nlm.nih.gov). Discrepancies in gene annotation between NCBI and Ensembl are listed in Table 1 where applicable. Primers for *TNFSF13B* amplify the longer transcript variant 1 that has been found in humans and other macaque species, but is formally not present in the rhesus macaque genome databases. Amplification efficiency was checked using serially diluted cDNA. Efficiencies between 94 and 98 % were regarded as sufficient for further analysis. Primer sequences for quantification of the housekeeping gene glyceraldehyde-3-phosphate dehydrogenase (*GAPDH)*, *MX1* and *CXCL10/IP-10* were taken from the report of Mussil *et al.* (2011). RNA levels of *GAPDH* were used as reference to calculate the relative expression levels of the target genes. All samples were run at least in duplicates. The results were analysed by using Sequence Detection Software (ABI). The relative expression levels of target genes were calculated as Δ*C*_t_ = mean *C*_t_ (target gene) − mean *C*_t_ (*GAPDH*). ΔΔ*C*_t_ was calculated as: Δ*C*_t_ of respective gene in CNAR(+) animals – Δ*C*_t_ of respective gene in CNAR(−) animals (Fig. 1) or control cultures (Fig. 4). Fold difference in gene expression was calculated as 2^−ΔΔ*C*_t_^. For other comparisons, relative gene expression was determined by using the formula rE=100×2^−ΔC_t_^. GraphPad Prism version 5 was used to plot the graphs.

**Table 1.** Rhesus macaque gene symbols and names, reference IDs and primer sequences

Gene symbol	Gene name	NCBI reference sequence/Ensembl ID	Primer sequence (5′ to 3′)
*CISH*	Cytokine inducible SH2-containing protein	NM_001258075.1	F: GGATGTGGTCAGCCTTGT R: CAGGCAGTGCTGGATCATTA
*CMA1*	Chymase 1, mast cell	NM_001194633.1	F: CACAGAATGCAAGCCACACT R: TGTCAGCACAAAGTTCCGTC
*CST6*	Cystatin E/M	XM_001111664.2	F: TACAACATGGGCAGCAACAG R: CCATCTCCATCGTCAGGAA
*DPP4*	Dipeptidyl-peptidase 4, CD26	NM_001039190.2	F: CAAATTGAAGCAGCCAGACA R: TCCCAGGACCATTGAGGTTA
*EPHB6*	EPH receptor B6	NM_001265762.1	F: ATGACCAGGCAGAAGACG R: CTCGCACCTGGAAACCATAG
*FAM26F*	Family with sequence similarity 26, member F	XM_001111520.2	F: TGTTGGGCTGGATCTTGATAG R: GCTCCTGTTCCAAATAGATTTTCC
*LOC721572/GAL-201*	Uncharacterized LOC721572/(galanin)	XR_091573.1/ENSMMUT00000033055	F: CCATGCCTGAGAACAATATCA R: GACCGCTCCATGTCTTCT
*GLUL*	Glutamate-ammonia ligase	NM_001266013.2	F: CCGGATTAGAAACCAAGCAT R: GCAGAAACCCAGAAGTGGTC
*GSTO1*	Glutathione *S*-transferase omega 1	XM_001099994.2	F: GGCTGGAAGCAATGAGGTTA R: GGCTGAGACTGTGGGATCTT
*MMP25*	Matrix metallopeptidase 25	XM_001091146.2/ENSMMUG00000009483	F: CTCACCCTGAATCCTCACTCA R: AACACCACGAGACAGTCAGCT
*PON3*	Paraoxonase 3	XM_001095770.2	F: TGCAGCCATTAGAACCTGAA R: AAGCCAGCCCACTAGGAAGT
*PSAT1*	Phosphoserine aminotransferase 1	XM_001101767.2	F: CCTGCTTATTTTGCCTTTGC R: TGTGTTCCCATGACTCCAGA
*RPL13*	60S ribosomal protein L13-like (LOC700603)	XM_002802597.1	F: CAAAGCCTTCGCTAGTCTCC R: TTTCAACATCCTGTTCTGCG
*SLAMF8-201*	SLAM family member 8	XM_001117299.1/ENSMMUT00000015880	F: GCAGATCCACACTGCTCAAA F: ATTGGTCTCACGGAAGCACT
*TNSF13B*	Tumour necrosis factor (ligand) superfamily, member 13b, transcript variant 1	XM_005586225.1	F: GCGATAAGTGGAGTCAGAGTTTC R: GCAAAAGGCAATGAAGGTTT

### IFN stimulation.

PBMCs from four SIV-infected LTNPs (plasma viral RNA copies <40 ml^−1^) were purified by density centrifugation using lymphocyte separation medium (PAA Laboratories), washed with PBS and incubated overnight at a concentration of 2.5×10^6^ cells ml^−1^ in RPMI complete medium at 37 °C. Cells were then plated in duplicate at a density of 2×10^6^ cells m in RPMI complete medium supplemented with IL-2 and rhesus IFN-α2 (5–10 ng ml^−1^; PBL Assay Science) or recombinant rhesus macaque IFN-γ (100 ng ml^−1^; R&D Systems) was added. In parallel, unstimulated PBMCs were used as controls. Cells were harvested by centrifugation at different time points and frozen in RNAprotect Cell Reagent (Qiagen). In a second set of experiments, PBMCs were activated by ConA (10 ng ml^−1^; SERVA Electrophoresis) for 8 h. After washing twice, cells were treated as described above. ΔΔ*C*_t_ was calculated by subtracting Δ*C*_t_ of the stimulated samples from Δ*C*_t_ of unstimulated controls. Fold difference in gene expression was calculated as 2^−ΔΔ*C*_t_^. Values at the start of experiments were set as 1.

### Viral RNA quantification.

Viral RNA was extracted from cell culture supernatant or plasma with the QIAamp Viral RNA Mini kit following the instructions of the manufacturer (Qiagen). Purified SIV RNA was quantified using RT-PCR (QuantiTect Probe RT-PCR kit; Qiagen) and the 7500 Real-Time PCR systems (Applied Biosystems) as described (Negri *et al.*, 2004).

### Quantification of IFN in plasma samples.

Plasma IFN-α and IFN-γ were quantified by ELISA using pan-specific antibodies for IFN-α (Mabtech) and rhesus macaque-specific antibodies for IFN-γ (Mabtech). Briefly, plasma was added to high binding microtitre plates (Greiner Bio-One) coated with the respective antibodies. Plasma IFNs were detected through the addition of biotinylated anti-IFN-γ monoclonal antibodies, streptavidin–horseradish peroxidase conjugate and tetramethylbenzidine substrate (Sigma-Aldrich). CXCL10/IP-10 was quantified using a cross-reactive human CXCL10/IP-10 ELISA kit (R&D Systems). Absorbance was measured at 405 nm with a 550 microplate reader (Bio-Rad).

### Statistical analysis.

Statistical analysis of differential gene expression of microarray data included ANOVA modelling, non-linear local regression normalization and component-wise *t*-tests. *P* values were adjusted for high-dimensional microarray data to control family-wise error rate or false discovery rate. Principal component analysis was applied to search for major differences (R-Bioconductor).

For comparison of two groups, the two-tailed nonparametric Mann Whitney *U* test was applied. For identification of potential correlation, Spearman's rank calculations were carried out.

Linear correlations were identified using Pearson correlation. The log-rank Mantel Cox test was used to calculate potential differences in the number of exposures required to infect all macaques.
