# Supplementary material for: Pre-infection transcript levels of FAM26F in peripheral blood mononuclear cells inform about overall plasma viral load in acute and post-acute phase after simian immunodeficiency virus infection
Source: J Gen Virol. 2016 Dec 15;97(12):3400–12. doi: 10.1099/jgv.0.000632 (PMC5203675; doi:10.1099/jgv.0.000632)
Supplement: Supplementary File 1 [file jgv-97-3400-s001.pdf]

Table S1:

De-regulated genes in CD8 cells from CNAR (+) versus CNAR(-) macaques

Genes investigated further are shaded in grey

Gene description	fold regulation	p-Value	human gene symbol or pseudogene	GenBank Accession no
**downregulated (>-2 fold)**				
cytochrome c oxidase subunit 8C	-7.46	0.0046	COX8C	NM_182971
cystatin E/M , CST6	-6.5	0.049	CST6	NM_001323
FAM26F	4.6	0.0368	FAM26F	XM_496823
PREDICTED: Homo sapiens similar to RIKEN cDNA A630077B13 gene; RIKEN cDNA 2810048G17 (LOC441168)	-4.6	0.0424	FAM26F	XM_496823
phosphoserine aminotransferase isoform 2 (LOC711679)	-4.29	0.0116	PSAT1	XR_012471
Macaca mulatta clone 65	-4.29	0.0259	NKG2-C	AF294890
Homo sapiens ribosomal protein L13	-4	0.0361	RPL13	NM_000977
cytokine inducible SH2-containing protein (CISH), transcript variant 1	-4	0.0268	CISH	NM_013324
Homo sapiens MCM4 minichromosome maintenance complex component 4	-3.48	0.0038	MCM4	NM_005914
TUBB6 tubulin, beta 6 class V, MGC4083	-3.48	0.0912	TUBB6	NM_032525
PREDICTED: Macaca mulatta protein disulfide isomerase-associated 4	-3.48	1.4e-05	PDIA4	XR_012082
dipeptidylpeptidase 4 (CD26, adenosine deaminase complexing protein 2)	-3.25	0.00037	DPP4	NM_001935
solute carrier family 39 (zinc transporter), member 8	-3.25	0.0552	SLC39A8, ZIP8	NM_022154
Homo sapiens SLAM family member 8	-3.25	0.00097	SLAMF8	NM_020125
adenylate cyclase 3	-3.25	0.00157	ADCY3	NM_004036
Homo sapiens neuroblastoma, suppression of tumorigenicity 1, transcript variant 1	-3.25	0.0485	NBL1	NM_182744
Homo sapiens catenin (cadherin-associated protein), alpha 1, 102kDa	-3.03	0.00242	CTNNA1	NM_001903
Homo sapiens LRRC71 leucine rich repeat containing 71, protein FLJ32884	-3.03	0.0114	C1orf92, LRRC71	NM_144702
Homo sapiens tumor necrosis factor (ligand) superfamily, member 13b	-3.03	0.000112	TNFSF13B	NM_006573
Homo sapiens glutamate-ammonia ligase (glutamine synthase)	-2.83	0.0071	GLUL	NM_002065
Homo sapiens flap structure-specific endonuclease 1	-2.83	0.0152	FEN1	NM_004111
Homo sapiens zinc finger protein 614	-2.83	0.00038	ZNF614	NM_025040
Homo sapiens sideroflexin 2 mRNA.	-2.83	0.0013	SFXN2	NM_178858
PREDICTED: Macaca mulatta similar to Dihydrofolate reductase (LOC702446)	-2.83	0.0182	pseudogene	XR_011953
Homo sapiens DPPA2 (LOC151871), developmental pluripotency associated 2	-2.83	0.00191	DPPA2	NM_138815
Homo sapiens aquaporin 3 (AQP3)	-2.83	5.87e-05	AQP3	NM_004925
Homo sapiens solute carrier family 24 (sodium/potassium/calcium exchanger), member 3	-2.83	0.00074	SLC24A3	NM_020689
Homo sapiens lipoma HMGIC fusion partner	-2.64	0.038	LHFP	NM_005780
Homo sapiens nerve growth factor receptor (TNFRSF16) associated protein 1	-2.64	0.0103	NGFRAP1	NM_014380
Homo sapiens B-cell CLL/lymphoma 7B (BCL7B), transcript variant 1	-2.64	0.00148	BCL7B	NM_001707
Homo sapiens S100 calcium binding protein A2	-2.64	0.00803	S100A2	NM_005978
Homo sapiens splicing factor proline/glutamine rich (polypyrimidine tract binding protein associated) (SFPQ)	-2.64	0.00528	SFPQ	NM_005066
Homo sapiens uracil-DNA glycosylase (UNG), nuclear gene encoding mitochondrial protein, transcript variant 1	-2.64	0.00904	UNG	NM_003362

PREDICTED: Macaca mulatta similar to Transcription factor Dp-1 (E2F dimerization partner 1) (DRTF1-polypeptide 1) (DRTF1)	-2.64	0.00571	TFDP1	XR_013654
Homo sapiens methionine sulfoxide reductase A	-2.64	0.00846	MSRA	NM_012331
Homo sapiens nerve growth factor receptor (TNFRSF16) associated protein 1, transcript variant 3	-2.64	0.00966	NGFRAP1	NM_014380
Homo sapiens glutathione S-transferase omega 1	-2.64	0.00765	GSTO1	NM_004832
PREDICTED: Macaca mulatta similar to ribonucleic acid binding protein S1 (predicted) (LOC702134)	-2.64	0.00439	pseudogene	XR_011614
Homo sapiens RNA binding motif (RNP1, RRM) protein 3 (), transcript variant 1	-2.46	0.032	RBM3	NM_006743
Homo sapiens DNA replication complex GINS protein PSF2	-2.46	0.0805	GINS2	NM_016095
Homo sapiens cell division cycle 25A (CDC25A), transcript variant 1	-2.46	0.0132	CDC25A	NM_001789
Homo sapiens MCM2 minichromosome maintenance deficient 2, mitotin (S. cerevisiae)	-2.46	0.0257	MCM2	NM_004526
Homo sapiens CHK1 checkpoint homolog (S. pombe) (CHEK1)	-2.46	0.0192	CHEK1	NM_001274
Homo sapiens Xg blood group (pseudoautosomal boundary-divided on the X chromosome) (XG)	-2.46	0.00371	XG	NM_175569
Homo sapiens solute carrier family 7 (cationic amino acid transporter, y+ system), member 5 (SLC7A5)	-2.46	0.00292	SLC7A5	NM_003486
Homo sapiens p21/Cdc42/Rac1-activated kinase 1 (STE20 homolog, yeast) (PAK1)	-2.46	0.00191	PAK1	NM_002576
Homo sapiens CSRP2 binding protein (CSRP2BP), transcript variant 1	-2.46	0.00083	CSRP2BP	NM_020536
Homo sapiens ribosomal protein S6 kinase, 90kDa, polypeptide 2 (RPS6KA2), transcript variant 1	-2.46	0.00018	RPS6KA2	NM_021135
Homo sapiens apoptosis, caspase activation inhibitor (AVEN)	-2.46	0.0117	AVEN	NM_020371
Homo sapiens KIAA1950 protein (KIAA1950),AMZ1 archaelysin family metallopeptidase 1	-2.46	0.00054	AMZ1	NM_133463
Homo sapiens MCM3 minichromosome maintenance deficient 3 (S. cerevisiae) (MCM3)	-2.46	0.075	MCM3	NM_002388
MMPL0007_B09 MMPL Macaca mulatta cDNA; similar to Human G protein gamma-11 subunit	-2.46	0.000724	GNG11	CB550573
Homo sapiens methylenetetrahydrofolate dehydrogenase (NADP+ dependent) 1-like (MTHFD1L)	-2.46	0.00462	MTHFD1L	NM_015440
Homo sapiens complement component 3 (C3)	-2.46	0.0774	C3	NM_000064
Homo sapiens thymosin, beta, identified in neuroblastoma cells (TMSNB)	-2.46	0.0611	TMSNB	NM_021992
Homo sapiens MGC4170 protein (MGC4170), N-acetylglucosamine-1-phosphate transferase, alpha and beta subunits	-2.46	0.00018	GNPTAB	NM_024312
Homo sapiens tumor necrosis factor (ligand) superfamily, member 10 (TNFSF10)	-2.46	0.00154	TNFSF10	NM_003810
Homo sapiens ubiquitin carboxyl-terminal esterase L1 (ubiquitin thiolesterase) (UCHL1)	-2.46	0.00054	UCHL1	NM_004181
Homo sapiens lipocalin 2 (oncogene 24p3) (LCN2)	-2.46	0.0485	LCN2	NM_005564
Homo sapiens matrix metalloproteinase 25 (MMP25), transcript variant 1	-2.46	0.0282	MMP25	NM_022468
PREDICTED: Macaca mulatta similar to activating transcription factor 7 interacting protein 2 (LOC710224)	-2.46	0.00536	ATF7IP2	XR_012838
Homo sapiens MCM10 minichromosome maintenance deficient 10 (S. cerevisiae) (MCM10), transcript variant 2	-2.46	0.083	MCM10	NM_018518
Homo sapiens cytoplasmic FMR1 interacting protein 2 (CYFIP2), transcript variant 2	-2.46	0.0095	CYFIP2	NM_001037332
Homo sapiens ribonucleotide reductase M2 polypeptide (RRM2)	-2.29	0.0617	RRM2	NM_001034
Homo sapiens phosphoglycerate dehydrogenase (PHGDH)	-2.29	0.022	PHGDH	NM_006623
Homo sapiens SCG5 secretogranin V (7B2 protein)	-2.29	0.00482	SCG5	NM_003020
Homo sapiens ANKRD35 ankyrin repeat domain 35	-2.29	0.031	ANKRD35	NM_144698
Homo sapiens tumor necrosis factor, alpha-induced protein 2 (TNFAIP2)	-2.29	0.0114	TNFAIP2	NM_006291
Homo sapiens v-myb myeloblastosis viral oncogene homolog (avian)-like 2 (MYBL2)	-2.29	0.0991	MYBL2	NM_002466
Homo sapiens cystathionase (cystathionine gamma-lyase) (CTH), transcript variant 1	-2.29	0.0292	CTH	NM_001902
Homo sapiens C16orf59 chromosome 16 open reading frame 59	-2.29	0.0904	C16orf59	NM_025108
Homo sapiens complement component (3d/Epstein Barr virus) receptor 2 (CR2)	-2.29	0.0127	CR2	NM_001006658

Homo sapiens pyrroline-5-carboxylate reductase 1 (PYCR1)	-2.29	0.0127	PYCR1	NM_006907
Homo sapiens TRIB2 tribbles pseudokinase 2	-2.29	0.00461	TRIB2	NM_021643
Homo sapiens nitric oxide synthase trafficking (NOSTRIN)	-2.29	0.006	NOSTRIN	NM_052946
Homo sapiens CD101 molecule	-2.29	1.5E+05	CD101	NM_004258
Homo sapiens TMEM155 transmembrane protein 155	-2.29	0.0142	TMEM155	NM_152399
Homo sapiens cyclin E2 (CCNE2), transcript variant 1	-2.29	0.0689	CCNE2	NM_057749
Homo sapiens solute carrier family 14 (urea transporter), member 1 (Kidd blood group) (SLC14A1)	-2.29	0.0376	SLC14A1	NM_015865
ILLUMIGEN_MCQ_42024 Katze_MMPB2 Macaca mulatta cDNA clone IBIUW:25077 5- similar to Bases 89 to 818 highly similar to human MGC12981 (Hs.104203)	-2.29	0.00297		CO648602
ILLUMIGEN_MCQ_28601 Katze_MMPB Macaca mulatta cDNA clone IBIUW:7533 5- similar to Bases 64 to 1022 highly similar to human APOL2 (Hs.398037)	-2.29	0.0852	APOL2	CN647493
Homo sapiens squalene epoxidase (SQLE)	-2.29	0.00222	SQLE	NM_003129
Homo sapiens helicase, lymphoid-specific (HELLS)	-2.29	0.0531	HELLS	NM_018063
Macaca mulatta epithelial-stromal interaction protein 1-like (LOC700208)	-2.29	0.0448	EPSTI1	
Homo sapiens cathepsin W (lymphopain) (CTSW)	-2.29	0.00487	CTSW	NM_001335
Homo sapiens jun dimerization protein 2 (JDP2)	-2.29	0.00009	JDP2	NM_130469
Homo sapiens PDIA3 protein disulfide isomerase family A, member 3	-2.29	0.00723	PDIA3	NM_005313
Homo sapiens Ras association (RalGDS/AF-6) domain family 5 (RASSF5)	-2.29	0.0178	RASSF5	NM_182663
Homo sapiens APBA2 amyloid beta (A4) precursor protein-binding, family A, member 2	-2.29	0.00506	APBA2	NM_005503
Homo sapiens DENND3 DENN/MADD domain containing 3	-2.29	0.00904	DENND3	NM_014957
Macaca mulatta CHAF1A chromatin assembly factor 1, subunit A (p150) [XR_014668]	-2.29	0.0877	pseudogene	XM_002801031.1
Homo sapiens thymidylate synthetase (TYMS)	-2.29	0.0602	TYMS	NM_001071
Homo sapiens HIRA histone cell cycle regulator	-2.29	0.0605	HIRA	NM_003325
Homo sapiens RRP1B ribosomal RNA processing 1B	-2.29	0.00646	RRP1B	NM_015056
Homo sapiens centromere protein H (CENPH)	-2.29	0.0519	CENPH	NM_022909
ILLUMIGEN_MCQ_33986 Katze_MMPL1 Macaca mulatta cDNA clone IBIUW:12562 5' similar to Bases 1 to 331 highly similar to human F5 (Hs.30054), mRNA sequence [CN803972]	-2.14	0.0418		
Homo sapiens WD repeat domain 5 (WDR5)	-2.14	0.00343	WDR5	NM_017588
ILLUMIGEN_MCQ_10044 Katze_MMPL2 Macaca mulatta cDNA clone IBIUW:9084 5' similar to Bases 7 to 588 highly similar to human TFRC (Hs.185726)	-2.14	0.00955		CN644277
Homo sapiens peptidylprolyl isomerase (cyclophilin)-like 1 (PPIL1)	-2.14	0.00723	PPIL1	NM_016059
Homo sapiens chromatin assembly factor 1, subunit B (p60) (CHAF1B)	-2.14	0.00371	CHAF1B	NM_005441
Macaca mulatta minichromosome maintenance deficient protein 5 (MCM5)	-2.14	0.0897	MCM5	XR_014169
Homo sapiens BCL2-associated athanogene 2 (BAG2)	-2.14	1.5E-05	BAG2	NM_004282
Macaca mulatta SYNE2 spectrin repeat containing, nuclear envelope 2	-2.14	0.026	SYNE2	XM_002805088.1
Homo sapiens phosducin-like 3 (PDCL3)	-2.14	0.00487	PDCL3	NM_024065
Homo sapiens serine hydroxymethyltransferase 1 (soluble) (SHMT1)	-2.14	0.0325	SHMT1	NM_004169
Homo sapiens NCAPH non-SMC condensin I complex, subunit H	-2.14	0.0806	NCAPH	NM_015341
Homo sapiens PNP purine nucleoside phosphorylase	-2.14	0.00168	PNP	NM_000270
Homo sapiens TSR1, 20S rRNA accumulation, homolog (S. cerevisiae)	-2.14	0.00013	TSR1	NM_018128
Homo sapiens solute carrier family 7 (cationic amino acid transporter, y+ system), member 5 (SLC7A5)	-2.14	0.00585	SLC7A5	NM_003486
Homo sapiens SEC31-like 2 (S. cerevisiae) (SEC31L2)	-2.14	0.063	SEC31B	NM_015490
Homo sapiens gamma-glutamyltransferase-like 4 (GGTL4)	-2.14	0.0188	GGTLC2	NM_080839
Homo sapiens TBC1 domain family, member 5 (TBC1D5)	-2.14	0.00211	TBC1D5	NM_014744
Macaca mulatta tripartite motif containing 5	-2.14	0.0131	TRIM5	AY523632
Homo sapiens family with sequence similarity 57, member B (FAM57B)	-2.14	0.00967	FAM57B	NM_031478

Homo sapiens gelsolin (amyloidosis, Finnish type) (GSN)	-2.14	0.0251	GSN	NM_000177
Homo sapiens carbonic anhydrase X (CA10)	-2.14	1.5E-05	CA10	NM_020178
Homo sapiens poly(A) binding protein, nuclear 1 (PABPN1)	-2.14	0.00326	PABPN1	NM_004643
Homo sapiens tryptophanyl-tRNA synthetase (WARS)	-2.14	0.0192	WARS	NM_004184
Homo sapiens RGL4 ral guanine nucleotide dissociation stimulator-like 4	-2.14	0.013	RGL4	NM_153615
Macaca mulatta succinate dehydrogenase cytochrome b560 subunit, mitochondrial-like	-2.14	8.24E-04	LOC699251	XR_010919.2
Homo sapiens GIPC PDZ domain containing family, member 1	-2.14	0.00432	GIPC1	NM_005716
ILLUMIGEN_MCQ_36452 Katze_MMBR Macaca mulatta cDNA clone IBIUW:13155 5' similar to Bases 1 to 676 highly similar to human D4S234E (Hs.79404), mRNA sequence	-2.14	0.0272		CN805363
Macaca mulatta mitochondrial aldehyde dehydrogenase 2 (ALDH2)	-2.14	0.0108		XR_012809
Homo sapiens zinc finger protein 541 (ZNF541)	-2.14	0.0867	ZNF541	NM_032255
Homo sapiens 6-phosphofructo-2-kinase/fructose-2,6-biphosphatase 3 (PFKFB3)	-2.14	0.0303	PFKFB3	[NM_004566
Homo sapiens integrin, beta 1 (fibronectin receptor, beta polypeptide, antigen CD29 includes MDF2, MSK12) (ITGB1)	-2.14	0.0151	ITGB1	NM_133376
Homo sapiens galactose mutarotase (aldose 1-epimerase) (GALM)	-2.14	0.0517	GALM	NM_138801
Homo sapiens gelsolin (amyloidosis, Finnish type) (GSN)	-2.14	0.0142	GSN	NM_000177
Homo sapiens suppressor of cytokine signaling 2 (SOCS2)	-2.14	0.05	SOCS2	NM_003877
Homo sapiens saccharopine dehydrogenase (putative) (SCCPDH)	-2.14	0.00427	SCCPDH	NM_016002

**Upregulated (>2fold)**	**fold upregulation**			
Homo sapiens URB1-AS1 URB1 antisense RNA 1 (head to head)	2.14	0.00724	URB1-AS1	NM_032910
Homo sapiens :HEATR9 HEAT repeat containing 9, C16H17orf66	2.14	0.0869	HEATR9	NM_152781
Homo sapiens histone 1, H3e	2.14	0.00038	HIST1H3E	NM_003532
Homo sapiens G protein-coupled receptor 78	2.14	0.0111	GPR78	NM_080819
Homo sapiens transient receptor potential cation channel, subfamily M, member 8	2.14	0.0912	TRPM8	NM_024080
Homo sapiens similar to RAB42, member RAS homolog family (LOC646996), Pseudogene	2.14	0.0291	pseudogene	XM_929975
Homo sapiens ENDOU endonuclease, polyU-specific, P11, PP11, PRSS26	2.3	0.0569	ENDOU	NM_006025
Homo sapiens galanin	2.3	0.0197	GAL	NM_015973
Homo sapiens protein FLJ20345, Meckel syndrome	2.46	0.0703	MKS1	NM_017777
Homo sapiens Rap guanine nucleotide exchange factor (GEF) 3.	2.64	0.0223	RAPGEF3	NM_006105
Homo sapiens histone 1, H4e	2.64	0.00455	HIST1H4E	NM_003545
Homo sapiens EphB6	2.64	0.00019	EPHB6	NM_004445
Homo sapiens major histocompatibility complex, class II, DP beta 1	2.83	0.00772	HLA-DPB1	NM_002121
Unknown Palmitoyltransferase	3.25	5.87E-05		

Table S2. Overview of the experiments

Experiment 1 (E1): AIDS vaccine experiment with three groups of 6 immunized animals and 6 monkeys serving as control (Tenbusch et al., 2012). Pre-infection RNA samples were available only for 14 macaques treated with an AIDS vaccine. Monkeys became infected through repeated i.r. low-dose challenge with increasing doses of SIVmac251.

Experiment 2 (E2): AIDS vaccine experiment consisting of two groups of 6 monkeys each, immunized with an experimental AIDS vaccine and 6 control macaques immunized with empty vectors. Pre-infection RNA samples were available for 11 immunized macaques and the 6 control macaques. Macaques were infected through a repeated i.r. low-dose challenges with 120 $TCID_{50}$ of SIVmac251. For stylistic reasons the 6 control macaques are termed as "unvaccinated" in the manuscript.

Experiment 3 (E3): Infection experiment consisting of 10 macaques (prospective study). Naïve macaques were initially exposed to low doses of SIVmac316 but did not become infected. After a waiting period of 18 weeks macaques were infected using the same procedure, virus (SIVmac251) and the same dose as macaques in E2.

	**E1**	**E2**	**E3**
Type of study	AIDS vaccine experiment	AIDS vaccine experiment	Infection experiment
No. of macaques treated with an AIDS vaccine	18	12	0
No. of RNA samples from immunized macaques before infection	14	11	0
No. of unvaccinated macaques	6	6	10
No of RNA samples unvaccinated from monkeys before infection	0	6	10
No. of RNA samples at wpi 2	23	0	10
No. of RNA samples at chronic phase (12, 24 wpi)	24,22	17	10
Infecting virus	SIVmac251	SIVmac251	SIVmac251
Route of infection	intrarectally	intrarectally	intrarectally
Quantities of inoculated virus per low-dose challenge	Escalating (30-120 $TCID_{50}$)	120 $TCID_{50}$	120 $TCID_{50}$

Figure S1

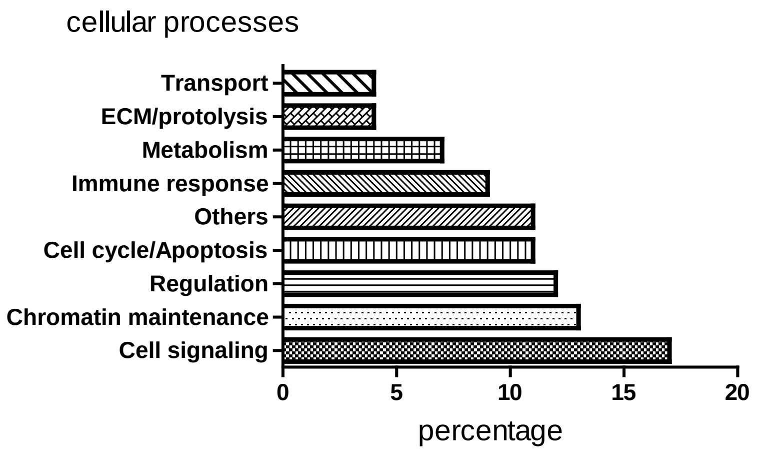

Fig. S1. De-regulated genes belong to diverse biological processes. The graph shows the percentages of the differentially regulated genes (differential expression >2 fold, p<0.1) detected through microarray study in $CD8^+$ T cells from CNAR(+) as compared to CNAR(-) monkeys and indicates their participation biological processes as defined by the Gene Ontology consortium.

Figure S2

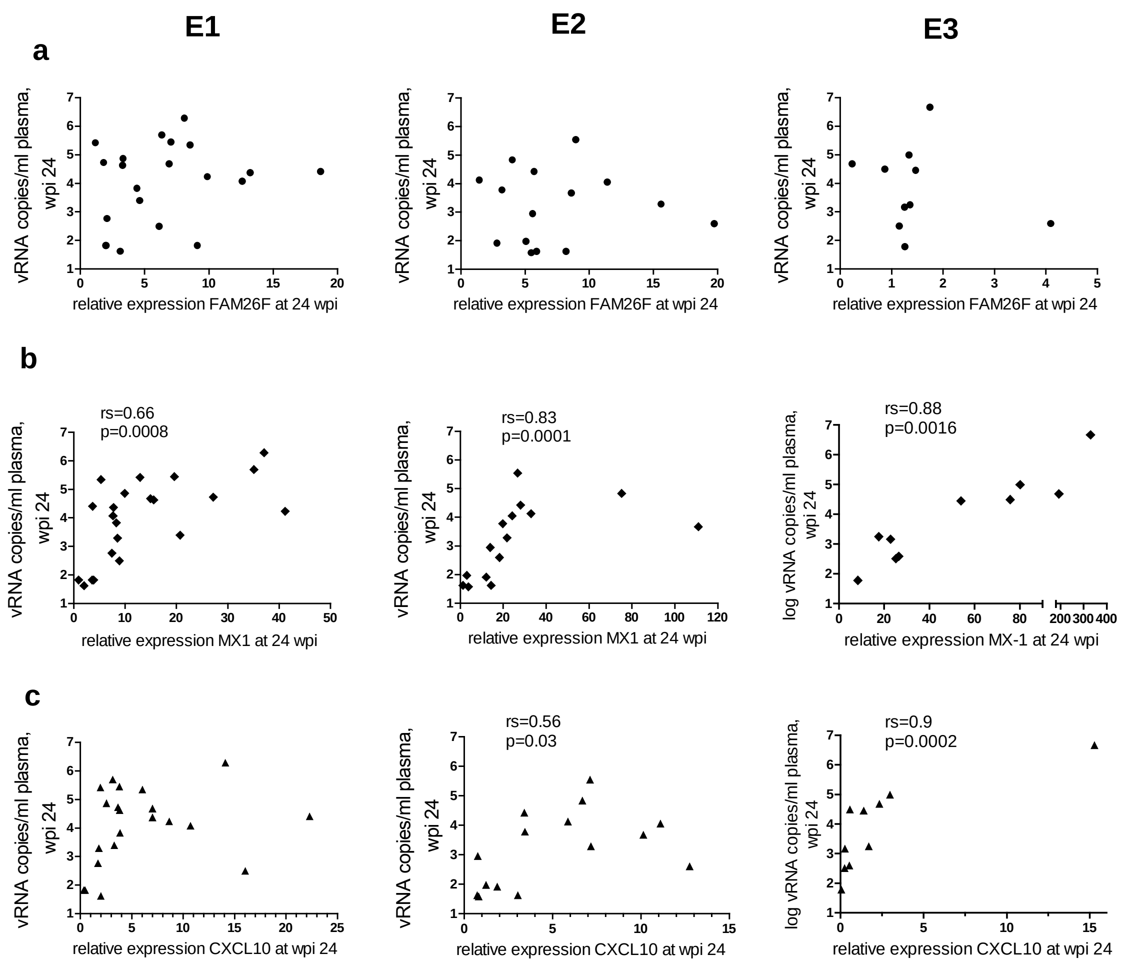

Fig. S2. Correlation of relative gene expression of *FAM26F*, *MX1* and *CXCL10* with viral load. Relative RNA levels of (a) *FAM26F*, (b) *MX1*, and (c) *CXCL10* was determined in PBMCs isolated at week 24 post infection in experiment 1-3 (E1-E3) and related to plasma viral load. Significant p values and Spearman rank correlation coefficient rs are indicated

Figure S3

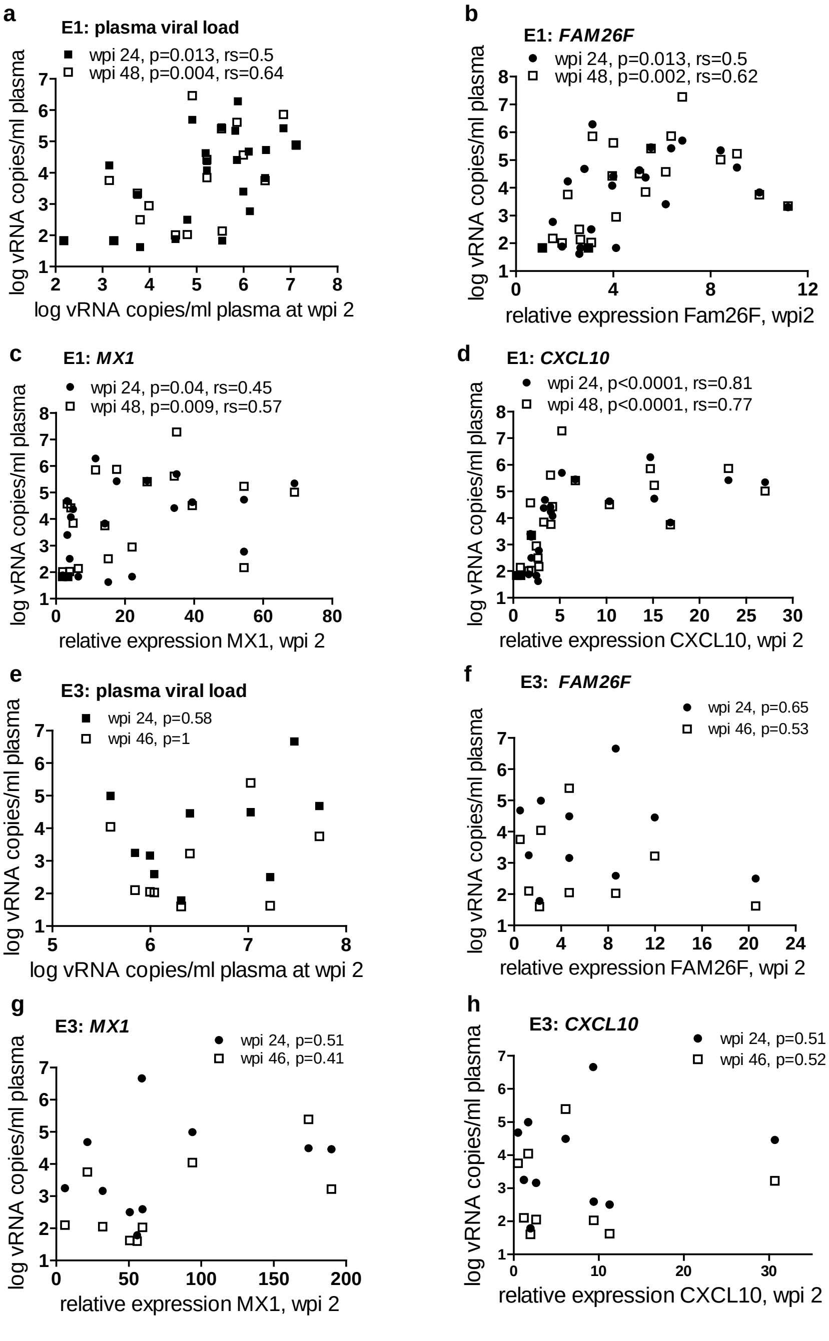

Fig. S3. Relation of RNA levels at 2 wpi with plasma viral load in the chronic phase of infection. To study if the RNA levels be an early predictor of viral load during the chronic phase of infection, potential correlations between transcript levels in PBMCs at wpi 2 and plasma viral load in chronic phase were investigated. In E1 (a) plasma viral RNA copies at wpi 2 and transcript levels of (b) *FAM26F*, (c) *MX1* and (d) *CXCL10* at 2 wpi were positively correlated with plasma viral RNA copies at wpi 24(n=23) and 48 (n=22). In E3 (n=10) (e) plasma viral RNA copies at wpi 2 and RNA levels of (f) *FAM26F*, (g) *MX1* and (h) *CXCL10* did not correlate with the chronic phase viral load at 24 (n=10) and 46 (n=9) wpi. P values are given in each figure, spearman´s rank order correlation coefficient rs is shown only when p values reached the level of significance ($p<0.05$).

Figure S4

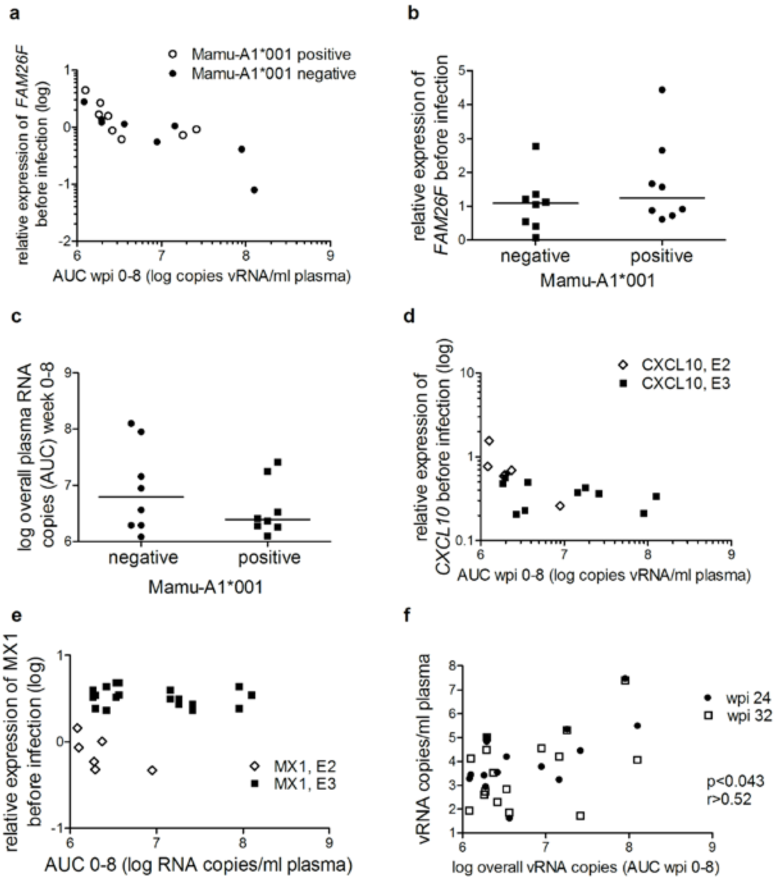

Figure S4. Supplementary figures for data from unvaccinated macaques (a) Relative RNA levels of *FAM26F* in PBMCs at day of first SIV-exposure correlated with overall plasma viral load wpi 0-8 in non-immunized macaques from E2 and E3 stratified according to presence of *Mamu-A1*001* (*Mamu-A1*001* positive: p=0.021, rs=0.81, n=8; *Mamu-A1*001* negative: p= 0.0004, rs=-0,9762, n=8).

(b) Pre-infection relative RNA levels of *FAM26F* in *Mamu-A1*001* negative and positive unvaccinated macaques (p=0.38, Mann Whitney test). Median is shown by a horizontal line.

(c) Overall plasma viral RNA copies from weeks 0-8 (AUC 0-8) in *Mamu-A1*001* negative and positive monkeys (p=0.44, Mann Whitney test). Median is shown by a horizontal line.

(d) Association of relative pre infection *CXCL10* transcript levels in PBMCs with overall plasma viral load of weeks 0-8 p.i. Neither overall viral load (AUC week 0-8) in E2 (p= 0.28) nor E3 (p=0.17) correlated with relative RNA of *CXL10* prior challenge. Combined data show an association (p=0.0017, rs=-0.72).

(e) Association of relative pre infection *MX1* transcript levels in PBMCs with overall plasma viral load of weeks 0-8 p.i. Neither overall viral load (AUC week 0-8) in E2 (p= 0.17) nor E3 (p=0.66) correlated with relative RNA of *MX1* prior challenge. Note, that relative RNA levels of E2 and E3 cannot be combined due to inherent differences in the PCR.

(f) The association of overall acute and post-acute phase plasma viral load (AUC wpi 0-8) with chronic phase plasma viral RNA copies (wpi 24 and 32) in unvaccinated SIV-infected macaques (n=16) is shown. Significance and Pearson correlation coefficient (r) for linear relationship is shown.

Figure S5

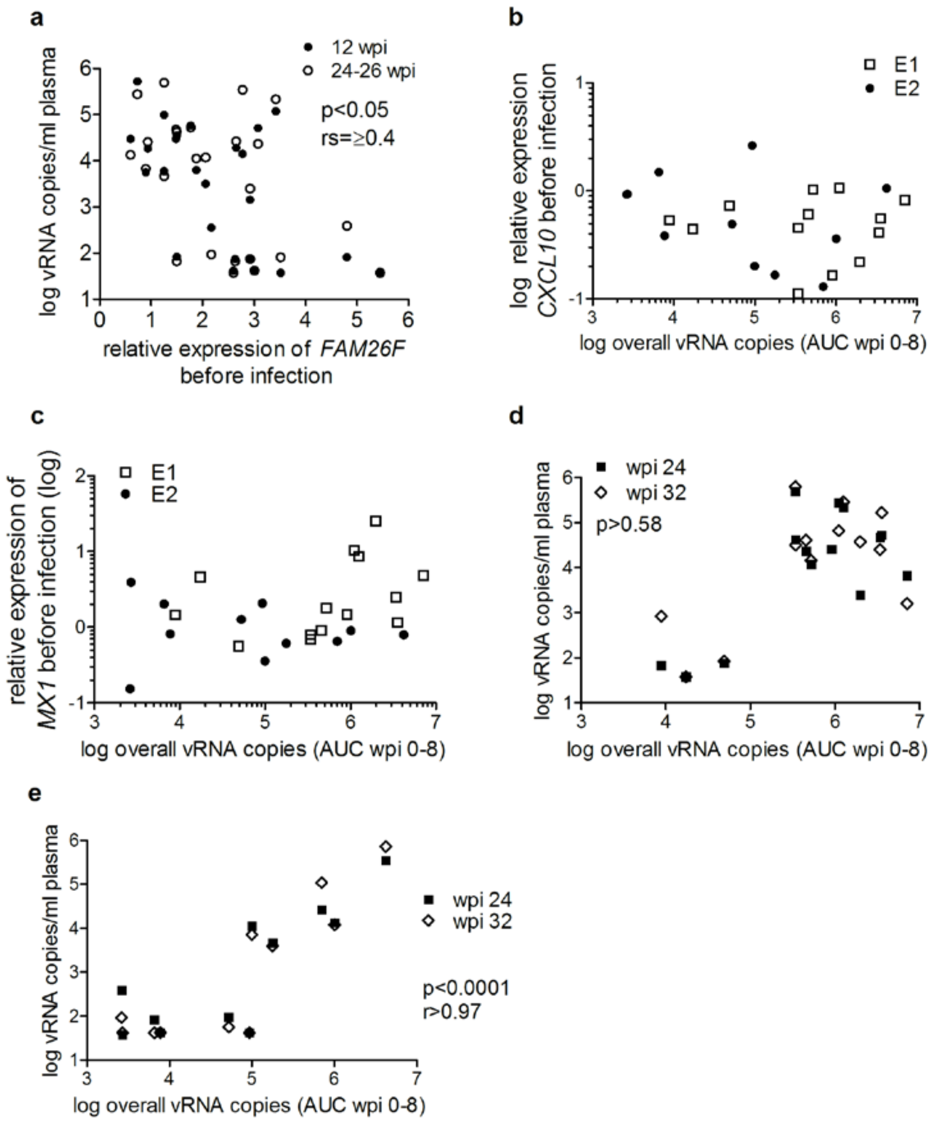

Fig. S5. Supplementary figures for data from macaques immunized with an AIDS vaccine. (a) Relative pre-infection expression of *FAM26F* in PBMCs correlated with plasma viral load at 12 wpi (n=25, rs=-0.49, p=0.012) and 24-26 wpi (n=25, rs=-0.4, p=0.046) in immunized macaques. Summarized p value and Spearman´s (rs) correlation coefficient are shown.

(b) Pre infection transcript levels of *CXCL10* in PBMCs from immunized macaques do not correlate with overall viral load from weeks 0-8 p.i. (E1: p=0.62, n=14; E2: p=0.25, n=11; combined: p=0.54, spearman rank correlation).

(c) Pre infection transcript levels of *MX1* in PBMCs from immunized macaques do not correlate with overall viral load from weeks 0-8 p.i. (E1: p=0.056, n=14; E2: p=0.54, n=11; combined: p=0.14, spearman rank correlation).

(d) Association of overall acute and post-acute phase plasma viral load (AUC wpi 0-8) with chronic phase plasma viral RNA copies (wpi 24 and 32) in immunized macaques from E1 (n=14), and

(e) from E2 (n=11). Significance and Pearson r are shown when applicable.
